# Social capital and oral health-related quality of life in pregnant women using public health services

**DOI:** 10.1590/1807-3107bor-2025.vol39.117

**Published:** 2025-11-07

**Authors:** Camila de Arruda Ribeiro PRATES, Jovito Adiel SKUPIEN, Bruno EMMANUELLI, Gabriela de ARAUJO, Daniele Prado ASSUMPÇÃO, Camila Silveira SFREDDO

**Affiliations:** (a) Universidade Franciscana – UFN, Postgraduate Program in Dental Sciences, Santa Maria, RS, Brazil.; (b) Universidade Federal de Santa Maria, Postgraduate Program in Dental Sciences, Santa Maria, RS, Brazil.; (c) Universidade Federal de Pelotas – UFPel, Postgraduate Program in Dentistry, Pelotas, RS, Brazil.

**Keywords:** Social Determinants of Health, Quality of Life, Oral Health, Pregnancy, Observational Study

## Abstract

Although social capital can significantly impact oral health-related quality of life (OHRQoL), evidence of this association remains limited during pregnancy. We assessed the association between individual social capital and OHRQoL among pregnant women in public health services. This cross-sectional study assessed a representative sample of pregnant women grouped into 25 Brazilian public health units in 2022. Sociodemographic, individual social capital (social networks and social support), and health behaviour characteristics were collected through a questionnaire. Participants were also clinically examined for gingivitis and dental caries experience. The outcome OHRQoL was assessed using the Oral Health Impact Profile (OHIP-14) questionnaire. Multilevel Poisson regression models with a hierarchical approach were used to assess the associations between social capital and overall OHIP-14 score. The results are presented as rate ratio (RR) and 95% confidence interval (95%CI). A total of 520 pregnant women were evaluated. Pregnant women with lower social support had higher overall OHIP-14 scores (RR: 1.21, 95%CI: 1.10–1.34). However, social networks were not associated with OHRQoL (p > 0.05). High number of children, lower household income and schooling factors, alcohol consumption before pregnancy, visiting a dentist for toothache, gingivitis, and dental caries experience were also associated with higher overall OHIP-14 scores (p < 0.05). Lower social support was associated with worse OHRQoL among pregnant women. These finding suggest that quality of personal resources was of greater relevance than social networks during pregnancy.

## Introduction

Social capital can be defined as characteristics of social organization, including networks, norms and trust, that facilitate the cooperation for the mutual benefit of both individuals and community.^1,2^ This concept is based on the consolidation of social networks and the establishment of reciprocity and reliability, which arise from relationships established in the social environment.^3^ In this context, social capital can be considered either an individual or contextual attribute despite the controversies over the concept.^2,4^ The individual dimension of social capital comprises the exclusive resources accessible through one’s social relationships, which facilitate the achievement of personal goals.^4,5^Frequently employed individual-level indicators in social capital research are social participation (e.g., organizational involvement and civic engagement), social networks, and social support.^5^ Social networks include relationships that a person has with family and friends as well as formal relationships with external groups, including voluntary associations, work, and other formal institutions.^6,7^ There are different social resources that are transmitted through a social network, including social support.^8^Social support, such as emotional and informational support, can be considered a qualitative dimension of the social networks to which an individual belongs.^5,9^While these concepts are related, they remain distinct in the literature, as social capital networks do not necessarily generate health-promoting social support.

There are several theories that establish a link between social capital and oral health.^2,10^ The behavioural theory recognizes that social capital can influence health through norms and informal social control and the dissemination of health-related knowledge,^2,10^ such as oral hygiene practices. The psychosocial theory emphasizes that high levels of social capital have stress-buffering effects on oral health via access to social support and feelings of safety, belonging, and coping.^2,10^ The influence of social capital through access to health services establishes that communities with civic engagement may have increased capacity to advocate for their interests, including access to high-quality health services.^2,10^ Moreover, social networks and social support encourage dental attendance.^11^

Social capital can influence oral health across different periods of life, including pregnancy.^6,12^ This period is characterized by biological and psychosocial changes as well as women’s adaptations to their motherhood and social environment.^13^ Moreover, oral diseases, such as gingivitis, periodontitis, and dental caries are prevalent and can negatively impact the oral health-related quality of life (OHRQoL) during pregnancy.^14^ OHRQoL is a multidimensional construct used to measure the impact of oral health conditions on everyday life and the individual’s perception of their life overall.^15^ This patient-reported outcome interacts with oral health conditions, general health, and social factors, such as social capital. In light of this scenario, the individual social capital can improve psychological distress and emotional well-being of pregnant women,^6^ and consequently, may have a positive effect on their oral health. Previous studies have evaluated the association between social capital and OHRQoL in pregnant and postpartum women.^12,16^ Lack of a family network and low levels of positive social interaction were associated with worse scores of OHRQoL during pregnancy and postpartum.^12,16^ However, the contextual social capital, based on day-to-day interaction between neighbours, did not significantly affect women’s OHRQoL during this period.^12^

There is limited evidence on the association between social capital and OHRQoL among pregnant women. Additionally, to the best of our knowledge, no prior study has examined the association of individual social capital on OHRQoL, adjusting for clinically assessed measures of oral health. The evaluation of social capital among pregnant women offers a valuable opportunity to comprehend the influence of social determinants during this crucial period of women’s lives. Moreover, efforts to enhance the health and quality of life of pregnant women hold the potential for positive effects on child health, as maternal behaviours serve as precursors to early childhood well-being. Therefore, this cross-sectional study aimed to explore the association between individual social capital and OHRQoL among pregnant women in public health services. We hypothesized that low individual social capital is associated with poorer levels of OHRQoL during pregnancy.

## Methods

This cross-sectional study is reported according to STROBE (Strengthening the Reporting of Observational Studies in Epidemiology) guidelines. Full description of the epidemiological survey is published elsewhere.^17^ The study protocol was approved by the Research Ethics Committee of the Federal University of Santa Maria (CAEE process: 54969222.9.0000.5346). All participants signed a free and informed consent form.

### Design and study population

This study recruted a representative sample of pregnant women who attended prenatal care at Primary Health Units administered by the National Health Care System [Sistema Único de Saúde (SUS)] in Santa Maria, an urban middle-sized city in the State of Rio Grande do Sul, Brazil. The city had an estimated population of 285,159 inhabitants,^18^ with 1,381 pregnant women registered in urban Primary Health Units, according to data from the city’s Department of Health in 2022. Prenatal care in Brazil is a considered a fundamental right and is ensured by the government through actions that improve the access to healthcare for pregnant women. This study was part of a larger project investigating various outcomes, including OHRQoL, dental service utilization, and dental caries. The sample size was determined based on one of the primary outcomes of the larger project (OHRQoL) using the following parameters: a 95% confidence interval (95% CI), a statistical power of 80%, an exposed-to-non-exposed ratio of 1:1, an effect size of 0.3 (small to medium effect),^19^ a design effect of 1.2, and a 20% nonresponse rate. Based on these criteria, the minimum required sample size was set at 507 pregnant women.

A two-stage sampling procedure was adopted. The primary sampling units were all 25 Primary Health Units of the urban area with prenatal care in Santa Maria. The selection process used an equal probability selection method (probability proportional to the unit size) to provide a representative sample from all Primary Health Units. The secondary sampling units were all pregnant women registered in the 25 Primary Health Units in 2022. Only pregnant women who were intellectually and physically capable of responding to the questionnaire were included. Women with high-risk pregnancies, noncognitive impairment, or those who were not able to read and understand Brazilian Portuguese were excluded from the analysis.

### Data collection

Data were collected from April to October 2022 through structured questionnaires and clinical oral examinations conducted with pregnant women at selected Primary Health Units.

### Oral health-related quality of life

OHRQoL was assessed using the Brazilian short version of the Oral Health Impact Profile (OHIP-14)^20^ at Primary Health Units through face-to-face interviews conducted by trained examiners prior to dental examinations. The OHIP-14 questionnaire consists of 14 questions grouped into seven domains: functional limitation, physical pain, psychological discomfort, physical disability, psychological disability, social disability and handicap. Responses were obtained on a Likert scale: 0 = “never”, 1 = “rarely”, 2 = “sometimes”, 3 = “repeatedly”, and 4 = “always”. The mean OHIP-14 scores were calculated by overall scores ranging from 0 to 56, with higher values indicating poorer OHRQoL.

### Individual social capital

Individual social capital was assessed based on social networks and social support. Social network aspects were captured through the frequency of relationships with friends and family, as well as participation in prenatal groups.^6,21^ The frequency of visiting relatives was measured by the question: “*Have you visited a relative/family member or has a relative/family member visited you in the last 12 months?*”. The options for the answer were: 0 = “yes, more than once a month”, 1 = “yes, at least once a month”, 2 = “yes, less than once a month” and 3 = “never or almost never”. Responses were categorized as “more frequent” (codes 0 and 1) and “less frequent” (codes 2 and 3).^17^ The social networks were also assessed through memberships in prenatal groups, as follows: “*Do you participate of group prenatal care for pregnant women?*” with the answers “yes” and “no”. Additionally, social support was assessed through the question: “If something unfortunate happened to you, would someone help you in this situation?” with the answers “yes” and “no”.^6,10^ This measure aimed to assess the individual’s perception of available support, rather than a network structure.

### Covariates

Sociodemographic and health-related behavior characteristics were collected through a structured questionnaire administered face-to-face by trained interviewers. Age was recorded in years and categorized as “adolescents” (13-19 years), “young adults” (20-24 years), and “adults” (25-50 years).^22^ Skin color was measured according to the Brazilian criteria^23^ and dichotomized into “white” and “non-white”. The category non-white included black, brown (pardos), indigenous, and yellow people. The number of children was categorized as 1, 2, and >3 based on prior criteria.^6^ Marital status was collected according to national criteria (“married”, “divorced”, “single”, and “widowed”),^23^ and categorized as “married” and “other”. Educational level was assessed through years of schooling and categorized as “≥ 8 years of schooling” and “< 8 years of schooling” (incomplete primary education). Monthly household income was measured in reals (R$) (Brazilian currency) and dichotomized by the median. Subsequently, the values were converted to their equivalent in the Brazilian Minimum Wage (BMW – approximately 200 USD in 2022), and the variable was categorized as “> 1.5 BMW” and “≤ 1.5 BMW”.

The health-related behaviours were smoking, alcohol consumption, and oral health habits. Smoking before pregnancy was collected by the following question: “Did you smoke before pregnancy?” with the answers “yes” and “no”.^6^ Alcohol consumption during pregnancy was assessed with the question: “Did you consume alcoholic beverages during pregnancy?” with response options “no” and “yes”. The women’s reasons to use dental service was collected through the question: “What was the reason for your last dental visit?”. Answers were categorized into “check-up/routine” and “toothache”.^24^ Toothbrushing frequency was categorized into “≥ 2 times a day” and “< 2 times a day”, in accordance with the recommendations of the American Dental Association.^25^

Full-mouth clinical examination was performed using standardized international criteria.^26^ Pregnant women were examined in a room with natural light using periodontal probes (“ball point”) and dental mirrors. Gingival bleeding was assessed in six sites per tooth according to the Community Periodontal Index (CPI),^26^ and was scored as 0 = “healthy” and 1= “bleeding”. Data on gingival bleeding were categorized as bleeding in “< 10% of sites (no gingivitis)” and bleeding in “≥ 10% of sites (gingivitis)”.^27^Dental caries experience was measured using the Decay, Missing, and Filled Teeth Index (DMFT),^26^ and categorized as “DMFT < 4” and “DMFT ≥ 4”, reflecting a more severe disease, in accordance with WHO guidelines.^28^ Prior to data collection, five examiners were trained and calibrated in a sample of women aged 18–45 years who were not included in the final analysis. The process included theoretical activities with discussion regarding the diagnostic criteria for all conditions and the examination of women. Inter- and intra-examiner agreement (Kappa statistics) for DMFT ranged from 0.76 to 0.88 and from 0.80 to 0.89, respectively.

### Statistical analysis

Data analysis was performed using Stata (StataCorp. 2014. Stata Statistical Software: Release 14.1. College Station, TX: StataCorp LP). The overall OHIP-14 score was the dependent variable. Descriptive analysis was performed for sociodemographic, psychosocial, behavioural, and clinical characteristics of the sample. Models were fitted by multilevel Poisson regression analysis to assess the association between individual social capital and OHRQoL. In the multilevel structure, pregnant women (level 1) were nested at Primary Health Units (level 2). The multilevel model used the scheme of fixed effect with random intercept. The results are presented as rate ratio (RR) and its respective 95% CI.

Four statistical models were tested according to a theoretical model:^1^ Model 1 (“null model”), an unconditional model estimated the proportion of variance for each level before the incremental introduction of individual independent variables; Model 2, sociodemographic variables; Model 3, comprised Model 2 plus individual social capital variables; Model 4, comprised Model 3 plus health-related behaviours variables; Model 5 (“full model”), comprised Model 4 plus clinical oral measures. Variables with p-value < 0.20 in the unadjusted analysis were considered in the multivariate models. Variables were retained in the analysis only if they had a p-value < 0.05 after adjustment. The quality of the fit of the models was evaluated using the deviance (− 2 log likelihood), and significant changes in the fitting of the models were assessed using the likelihood ratio test.

## Results

Initially, 558 pregnant women were invited to participate in the study. The response rate was 93%. The reasons for refusals included time shortage and lack of interest in participating in the study (n = 29), not speaking Portuguese (n = 1), shame/fear (n = 4), cognitive problems (n = 2), and testing positive for COVID-19 (n = 2). The final sample was 520 women nested in 25 Primary Health Units. Most pregnant women were in the third trimester (41.3%).

Sociodemographic, social capital, health-related behaviours, and clinical oral health characteristics of the participants are summarized in Table 1. Participants were predominantly adults, white, and had only one child. The mean household income was R$ 2017.7, and most women had at least 8 years of schooling. In relation to individual social capital, pregnant women reported frequent visits to family members and having social support. However, the majority of participants did not participate in group prenatal care for pregnant women. The prevalence of gingivitis and severe dental caries were 44.3% and 40.9%, respectively. The overall CPQ11-14 score ranged from 0 to 47 [mean = 9.9; standard deviation (SD) = 8.9].

**Table 1:** Sample distribution according to sociodemographic, social capital, behavioural, clinical, oral health characteristics of pregnant women (n= 520).

Variables	n (%)*
Sociodemographic	
Age	
Adolescent	66 (12.7)
Young adult	151 (29.1)
Adult	302 (58.2)
Skin colour	
White	313 (60.2)
Non-white	207 (39.8)
Number of children	
1	214 (41.1)
2	146 (28.1)
> 3	160 (30.8)
Marital status	
Married	163 (31.3)
Other	357 (68.7)
Educational level (years)	
≥ 8	372 (71.5)
< 8	148 (28.5)
Monthly household income (BMW)	
> 1.5	265 (51.0)
≤ 1.5	255 (49.0)
Individual social capital	
Frequency of relative/family visit	
More frequent	401 (77.1)
Less frequent	119 (22.9)
Membership in prenatal group	
Yes	46 (8.9)
No	473 (91.1)
Social support	
Yes	483 (92.9)
No	37 (7.12)
Health-related behaviours	
Smoking before pregnancy	
No	385 (74.0)
Yes	135 (26.0)
Alcohol consumption	
No	448 (86.1)
Yes	72 (13.9)
Reason for visiting a dentist	
Check-up/routine	380 (73.9)
Toothache	134 (26.1)
Tooth brushing frequency (daily times)	
< 2	492 (94.6)
≥ 2	28 (5.4)
Clinical oral health measures	
Gingivitis	
No (< 10% of sites)	289 (55.7)
Yes (≥ 10% of sites)	230 (44.3)
Severity of dental caries experience	
DMFT< 4	307 (59.0)
DMFT ≥ 4	213 (40.9)
Overall OHIP-14 [Mean (SD)]	9.9 (8.9)

BMW: Brazilian minimum wage (1 BMW was approximately equivalent to 200 USD in 2022); DMFT: Decay, Missing, and Filled Teeth Index; OHIP-14: Oral Health Impact Profile; SD: standard deviation. *Values less than 520 are due to missing data.

Table 2 presents the unadjusted associations between individual social capital variables and overall CPQ11-14 scores. Pregnant women who reported lower frequency of family visits and lack of social support showed higher overall OHIP-14 scores (p < 0.01). The overall OHIP-14 score was also associated with age, number of children, schooling, and household income (p < 0.01). Moreover, smoking, visiting a dentist due to toothache, and adverse oral health conditions were related to OHIP-14 scores (p < 0.01).

**Table 2:** Unadjusted associations between independent and overall OHIP-14 scores, determined using multilevel Poisson regression.

Variables	RR (95%CI)	p-value
Sociodemographic		
Age		0.05
Adolescent	1	
Young adult	0.89 (0.81-0.98)	
Adult	0.90 (0.82-0.97)	
Skin colour		0.12
White	1	
Non-white	1.04 (0.98-1.10)	
Number of children		< 0.01
1	1	
2	1.03 (0.96-1.11)	
> 3	1.46 (1.37-1.56)	
Marital status		0.66
Married	1	
Other	1.01 (0.95-1.07)	
Educational level (years)		< 0.01
≥ 8	1	
< 8	1.36 (1.28-1.44)	
Monthly household income (BMW)		< 0.01
> 1.5	1	
≤ 1.5	1.24 (1.17-1.32)	
Individual social capital		
Frequency of relative/family visit		< 0.01
More frequent	1	
Less frequent	1.11 (1.04-1.19)	
Membership in prenatal group		0.27
Yes	1	
No	1.06 (0.95-1.18)	
Social support		< 0.01
Yes	1	
No	1.44 (1.31-1.58)	
Health-related behaviours		
Smoking before pregnancy		< 0.01
No	1	
Yes	1,15 (1.08-1.23)	
Alcohol consumption		0.46
No	1	
Yes	1.03 (0.95-1.11)	
Reason for visiting a dentist		< 0.01
Check-up/routine	1	
Toothache	1.60 (1.51-1.70)	
Tooth brushing frequency (daily times)		0.06
< 2	1	
≥ 2	1.11 (1.00-1.25)	
Clinical oral health measures		
Gingivitis		< 0.01
No (< 10% of sites)	1	
Yes (≥ 10% of sites)	1.18 (1.12-1.26)	
Severity of dental caries experience		< 0.01
DMFT< 4	1	
DMFT ≥ 4	1.26 (1.19-1.33)	

RR: rate ratio; CI: confidence interval; BMW: Brazilian minimum wage (1 BMW was approximately equivalent to 200 USD in 2022); DMFT: Decay, Missing, and Filled Teeth Index.

The multilevel Poisson regression models are shown in Table 3. In Model 2, pregnant women with a higher number of children (RR = 1.54; 95%CI: 1.43–1.66), lower schooling (RR = 1.18; 95%CI: 1.10–1.26), and lower household income (RR = 1.11; 95%CI: 1.04–1.18) had higher overall CPQ11-14 scores. Pregnant women classified as young adults (RR = 0.84; 95%CI: 0.76–0.93) and adults (RR = 0.75; 95%CI: 0.68–0.83) had better OHRQoL compared to adolescent pregnant women (Model 2). Individual social capital variables were inserted in model 3. In this model, lack of social support was associated with poorer OHRQoL in pregnancy (RR = 1.21, 95%CI: 1.10-1.34). Model 4 included health-related behaviours. The overall CPQ11-14 scores were significantly higher among pregnant women who smoked before pregnancy (RR = 1.09; 95%CI: 1.03–1.17) and visited a dentist due to toothache (RR = 1.53; 95%CI: 1.44–1.62). The final model (Model 5) included clinical oral measures and revealed that pregnant women with gingivitis (RR = 1.10; 95%CI: 1.03–1.17) and greater severity of dental caries (RR= 1.24; 95%CI: 1.17–1.32) had higher overall OHIP-14 scores. Table 3 also demonstrated that model 5 fitted better than the previous models as a reduction on the deviance was observed (likelihood ratio test; p ≤ 0.05).

**Table 3:** Adjusted associations between independent variables and overall OHIP-14 scores, determined using multilevel Poisson regression.

Variables	Model 1^a^ (“null”)	Model 2^b^	Model 3^c^	Model 4^d^	Model 5^e^ (“full”)
	RR (95%CI)	RR (95%CI)	RR (95%CI)	RR (95%CI)	RR (95%CI)
Fixed component intercept	9.71 (8.81–10.69)	8.93 (7.89–10.10)	8.93 (7.89–10.10)	7.32 (6.42–8.34)	6.95 (6.11–7.92)
Age					
Adolescent		1	1	1	1
Young adult		0.84 (0.76–0.93)	0.84 (0.76–0.93)	0.84 (0.76–0.93)	0.84 (0.76–0.93)
Adult		0.75 (0.68–0.83)	0.75 (0.68–0.83)	0.75 (0.68–0.83)	0.75 (0.68–0.83)
Skin colour		*	*	*	*
White					
Non-white					
Number of children					
1 child		1	1	1	1
2 children		1.10 (1,02–1,19)	1.10 (1,02–1,19)	1.10 (1,02–1,19)	1.10 (1,02–1,19)
> 3 children		1.54 (1.43–1.66)	1.54 (1.43–1.66)	1.54 (1.43–1.66)	1.54 (1.43–1.66)
Marital status		*	*	*	*
Married					
Other					
Educational level					
≥ 8 years		1	1	1	1
< 8 years		1.18 (1.10–1.26)	1.18 (1.10–1.26)	1.18 (1.10–1.26)	1.18 (1.10–1.26)
Monthly household income					
> 1.5 BMW		1	1	1	1
≤ 1.5 BMW		1.11 (1.04–1.18)	1.11 (1.04–1.18)	1.11 (1.04–1.18)	1.11 (1.04–1.18)
Frequency of relative/family visit			*	*	*
More frequent					
Less frequent					
Membership in prenatal group			*	*	*
Yes					
No					
Social support					
Yes			1	1	1
No			1.21 (1.10–1.34)	1.21 (1.10–1.34)	1.21 (1.10–1.34)
Smoking before pregnancy					
No				1	1
Yes				1.09 (1.03–1.17)	1.09 (1.03–1.17)
Alcohol consumption				*	*
No					
Yes					
Reason for visiting a dentist					
Check-up/routine				1	1
Toothache				1.53 (1.44–1.62)	1.53 (1.44–1.62)
Tooth brushing frequency (daily)				*	*
< 2 times					
≥ 2 times					
Gingivitis					
No (< 10% of sites)					1
Yes (≥ 10% of sites)					1.10 (1.03–1.17)
Severity of dental caries experience					
DMFT< 4					1
DMFT > 4					1.24 (1.17–1.32)
Random effect					
Deviance (− 2 loglikelihood)	5,772.81	5,502.65	5,488.23	5,193.42	5118.18

RR: rate ratio; CI: confidence interval; BMW: Brazilian minimum wage (1 BMW was approximately equivalent to 200 USD in 2022); DMFT: Decay, Missing, and Filled Teeth Index. ^a^Model 1: null model, represents unconditional model. ^b^Model 2: mutually adjusted for sociodemographic variables. ^c^Model 3: mutually adjusted for sociodemographic and social capital variables. ^d^Model 4: mutually adjusted for sociodemographic, social capital variables, and health-related behaviours. ^e^Model 5: full model adjusted for sociodemographic, social capital variables, health-related behaviours, and clinical oral health measures. *Variables not included in the final multiple models after the adjustment.

## Discussion

The present study supports the hypothesis that low individual social capital is associated with poorer levels of OHRQoL during pregnancy. Pregnant women with lower social support had higher overall OHIP-14 scores. However, social networks, including the frequency of visits from relatives and family and membership in a prenatal group, were not related to OHRQoL in pregnant women. Our findings also suggest that a higher number of children, lower household income and schooling factors, alcohol consumption before pregnancy, visiting a dentist due to toothache, gingivitis, and dental caries experience are associated to poor OHRQoL during pregnancy.

Our findings demonstrated that pregnant women with lack of social support presented higher overall OHIP-14 in accordance with previous studies.^12,16^ The literature has also demonstrated that lack of social support has been associated with poor health-related quality of life^9^ and self-rated health,^14,29^ anxiety symptoms, and lower levels of life satisfaction among pregnant women.^30^ Social support refers to a network of formal and informal relationships through which individuals receive emotional, material, appraisal, or informational support to cope with distressing situations in life.^5,9^ The main theoretical explanation for the influence of social support on OHRQoL is the psychosocial theory.^29^ According to this theory, social support has a stress-buffering effect and contributes to the well-being of individuals by enhancing their ability to cope with stress.^2^ Therefore, social support influences how pregnant women perceive their lives and health, consequently impacting their OHRQoL.^12^ On the other hand, social support can also have a positive impact on health by encouraging healthy behaviours, such as attending dental health services.^2^

However, social networks were not associated with OHRQoL. Some studies have shown that low social networks and social capital is related with poor OHRQoL,^12,31^ while others did not find this association.^24^ Methodological differences in measuring social capital may help to explain these contrasting findings. Moreover, our findings suggest that quality of personal social resources (social support) of pregnant women may be more important for OHRQoL than the social networks, including relationship with family and participation in prenatal group. A previous study assessed the differences in OHRQoL between women connected to either predominantly home-based and work-based social networks and revealed the modifying effect of social support.^16^ Women who were in paid employment outside the home and had social supports had better OHRQoL than those with home-based social network.^16^ However, employment is not sufficient for reducing the effects on oral health. High social support combined with work-based social network is necessary for a better OHRQoL in pregnant and postpartum women.^16^ During pregnancy, emotional and material support from friends, close people, or families can be more important to improve women’s well-being and satisfaction with life.^17,32^

Sociodemographic characteristics were also associated with OHRQoL in pregnant women. Pregnant adolescents and those with a higher number of children exhibited higher overall OHIP-14 score. It has been shown that younger mothers have decreased overall quality of life^9,33^ and OHRQoL^34^ compared to older mothers. Adverse medical conditions and social issues such as school absenteeism, social isolation, and unemployment experienced during pregnancy can explain the poor quality of life observed in pregnant adolescents.^33,35^ Having many children was also related to poor self-rated health^16^ and OHRQoL^34^ among pregnant and postpartum women in accordance with previous studies. A possible explanation is that a higher number of children increased the workload and affect mental and physical health, oral hygiene behaviours, and quality of life.^33,34^ Our findings also demonstrated that low household income and low schooling impacted negatively the women’ OHRQoL in accordance with previous studies.^16,17,34^ It is recognized that people of lower socioeconomic status have worse OHRQoL in all age groups, irrespective of the socioeconomic indicator used.^36^ Socioeconomic inequalities reflect lack of material resources and access to health services, producing higher levels of disease.^1^ In addition, emotional and social meanings of inequalities for women’s own lives can impact coping behavior and healthy habits, impacting OHRQoL.^17,24^

Health-related behaviours were also associated with OHRQoL among pregnant women in our study. Pregnant women who smoked before pregnancy and visited a dentist due to toothache had the worst OHRQoL. Smoking is one of the major environmental risk factors for periodontal diseases.^37^ Qualitative changes in the subgingival biofilm, damage to inflammatory and immune responses, and impaired healing capacity can explain the effect of smoking on periodontal tissues.^37,38^ Therefore, negative effects of smoking on oral health can lead to poor OHRQoL during pregnancy.^6^ Although smoking during pregnancy was not assessed in our study, socioeconomic inequalities influence cigarette consumption, and thus, women may smoke during pregnancy, affecting their oral health.^39^ In addition, previous studies demonstrated the negative impact of toothache on OHRQoL.^21,24^ Toothache affects physical status, psychological well-being, and social interactions of individuals, impacting the OHRQoL.^21,24^

Our study also demonstrated that pregnant women with gingivitis and severe dental caries had poor OHRQoL. It is recognized that poor oral health negatively impacts OHRQoL across different populations.^21,24,36^ Gingivitis can affect the functional and discomfort domains during pregnancy.^14^ Moreover, self-reported symptoms of gingivitis, such as swollen and sore gums, were associated with an increased impact on OHRQoL by psychosocial and social aspects.^40^ A previous study has also shown the negative impact of dental caries on OHRQoL among adults.^41^ Individuals with severe dental caries can experience dental pain and impaired chewing,^21,24^ which may negatively impact their self-perception of oral health and quality of life during pregnancy.

The present study has limitations that should be addressed. The major shortcoming is that social capital was measured using indirect indicators of social networks and social support rather than standardized questionnaires, which may not fully capture the complexity of social capital. However, there are divergent theories and measurements of social capital in the literature.^36^ Social capital is a complex, multidimensional construct that cannot be directly measured, as there is no universally accepted tool to assess it.^5^ Our study used individual social capital variables based on indicators of social capital described in previous studies.^6,10,12^ While this approach may not capture all the dimensions of social capital, it is commonly used in public health research due to its practicality and relevance for identifying key areas of intervention. However, we acknowledge this limitation and suggest that future studies consider multidimensional tools for a more comprehensive evaluation. Although we recruited only pregnant women who were enrolled in the public healthcare service, participants from different socioeconomic status were selected in all Primary Health Units and neighbourhoods of the city, allowing to address the social gradient in health. Another limitation of this study is that only the presence of gingivitis was assessed and the clinical periodontal parameters necessary to classify cases of periodontitis were not included. This decision was due to practical limitations, as the oral evaluations were conducted in a room with natural light and without a dental chair. This made it difficult to assess subgingival parameters such as clinical attachment level required for diagnosing periodontitis according to the current classification. However, it is important to note that gingivitis, a highly prevalent periodontal disease during pregnancy,^37^ was measured and included in the final model.

This study has also some strengths. Social capital has been highlighted as one of the main determinants of health according to the WHO. Our study assessed the association between social capital and OHRQoL during a crucial period in women’s lives, providing evidence for health promotion in this population. This knowledge may provide policy makers with useful insights into the importance of social relationships for the health and OHRQoL of individuals during pregnancy. In addition, a full-mouth clinical evaluation was performed in all pregnant women included in the present study. Use of dental services and oral health-related behaviours were also collected to validate the subjective oral health measures. Attending a dentist due to toothache and poor oral health, including periodontal disease and dental caries experience, may be important factors influencing the association between social capital and OHRQoL.^21,24^ Therefore, it is essential to include them in the multivariate model. Finally, assessing patient-reported outcomes such as OHRQoL captures perceptions of social interactions and satisfaction with oral health.

The findings of our study emphasize the role of low individual social capital as a determinant of poorer OHRQoL in pregnant women and highlight the need to integrate social support strategies into prenatal and oral health programs. Healthcare professionals, including dentists and prenatal care providers, should consider assessing social support levels and implementing interventions that strengthen social support. This could involve adopting multidisciplinary approaches, such as partnerships between dental and social services, to provide comprehensive care for pregnant women at higher risk of poor OHRQoL. Such integrated efforts may contribute to improved health outcomes and overall well-being during pregnancy.

## Conclusions

Our findings indicate that pregnant women with lower social support have higher overall OHIP-14 score, emphasizing the role of social support as a key determinant of OHRQoL during pregnancy. Given the study’s focus on individual social capital, it is important to clarify that social support, as a qualitative dimension of social networks, plays a crucial role in influencing health outcomes. Health strategies aimed at strengthening social support—particularly emotional and informational support—should be implemented to improve the quality of life of pregnant women.

## Data Availability

After publication the data will be available on demand to the authors - condition justified in the manuscript.
